# Accountability for tackling childhood obesity: insights from local councillors in England

**DOI:** 10.1057/s41271-025-00573-x

**Published:** 2025-07-25

**Authors:** Ravita Taheem, Kathryn Woods-Townsend, Wendy Lawrence, Janis Baird, Keith M. Godfrey, Mark A. Hanson

**Affiliations:** 1 NIHR Southampton Biomedical Research Centre, University Hospital Southampton, NHS Foundation Trust, Southampton, UK; 2 Southampton City Council, Civic Centre, Southampton, UK; 3 Healthcare Enterprise and Innovation, Faculty of Medicine, University of Southampton, Southampton, UK; 4 Medical Research Council Lifecourse Epidemiology Centre, University of Southampton, Southampton, UK; 5 Primary Care, Population Science and Medical Education, Faculty of Medicine, University of Southampton, Southampton, UK; 6 NIHR Applied Research Collaboration Wessex, Southampton Science Park, Innovation Centre, 2 Venture Road, Chilworth, Southampton, UK; 7 Institute of Developmental Sciences, University of Southampton, Southampton, UK; 8 Institute of Developmental Sciences, Human Development and Health Faculty of Medicine, University Hospital Southampton, University of Southampton, Tremona Road, Southampton, SO16 6YD UK

**Keywords:** Governance, Local government, Childhood obesity, Public health, Health promotion

## Abstract

Tackling the complex drivers of childhood obesity requires action across sectors and at all levels of government. Elected officials in local government can influence policies targeting communities to prevent childhood obesity, but little is known about their views on local government accountability for tackling the issue. Accountability is the obligation to justify actions on a topic and it could strengthen policy implementation. A qualitative study was conducted involving semi-structured interviews with sixteen Southampton City Council local government councillors. Factors limiting accountability included low citizen engagement, the lack of a national mandate to support local action and unachievable targets. Factors that improved accountability included setting a local mandate, public health officers proactively keeping the issue on the agenda and oversight from other system leaders. The findings from this study inform how public health officers and other stakeholders can work within the local system to progress childhood obesity prevention policies.

## Key messages

- Public health officers should proactively use the local democratic infrastructure to improve accountability for tackling childhood obesity.
- Political oversight for tackling childhood obesity could be improved by engaging local system leaders with the issue.

## Introduction

Childhood obesity is a major health challenge with 159 million school-aged children and adolescents living with obesity worldwide in 2022 [1]. The National Child Measurement Programme in England has shown progressively widening disparities in the prevalence of childhood obesity over the last decade, with children living in the most disadvantaged areas now twice as likely to be obese compared to those living in the least disadvantaged areas [2].

Obesity in children and adolescents often precedes obesity in adulthood, increasing the likelihood of many disorders including type 2 diabetes, cardiovascular disease, asthma or wheezing and certain cancers [3–5]. Obesity emerges from a complex system of interactions between factors at the biological, socio-behavioural, political, environmental and economic levels [6]. These are driven by political policies which shape the environment and social conditions to either weaken or reinforce healthy behaviours [7–9].

Policies are used by governments to influence change or frame a problem that has been recognised by the public or policy-makers [10]. Various policy tools are available to governments, such as policies for giving information to the public, or enactment of legislation disincentivising a behaviour through fining individuals [10]. In England the *Childhood Obesity Action Plan 2018,* announced the goal of halving childhood obesity by 2030 through a range of national government-led actions including restrictions on price promotions of unhealthy food and drink products, alongside encouraging local authorities to use their existing powers for reducing their childhood obesity levels [11]. In England a local authority-led whole systems approach for tackling obesity or childhood obesity is recommended, whereby a network of internal and external actors identify the underlying system behaviours and enacts a variety of policies and interventions to address the issue [12].

Existing research has described factors that encourage and discourage support for childhood obesity prevention policies at a national level in the United States (US). Aligning with policy-maker priorities and community-led collaborations can encourage policy-maker support for childhood obesity prevention policies. Conversely, lack of funding, industry opposition and an unfavourable political environment can discourage policy-makers from supporting childhood obesity prevention policies [13].

The United Kingdom (UK) government has devolved many public health responsibilities to local government, including urban environment, local transport and licencing powers which could promote a healthier weight environment. These provide important opportunities to influence policies that could reduce childhood obesity rates. Constrained local government budgets, competing priorities and lack of strategic oversight can, however, stall progress [14]. Democratically elected councillors in England can set budgets and inform policy development and their support for tackling childhood obesity may bolster investment and buy-in from other departments and sectors [15–17]. However, there is currently little research examining how councillors see their role and whether they perceive an obligation to address this issue [18].

Given that tackling childhood obesity is a public health priority for many local authorities, councillors’ views on local government’s accountability for tackling childhood obesity may have implications for local childhood obesity prevention policies. Accountability is where an individual (agent) accepts the requirement to provide information and justify their actions on an issue to a principal or a forum [16, 18]. This obligation to report to another individual may be vertical (hierarchical), or horizontal accountability among agencies or to the public on a voluntary basis [16]. In this study, we invited local councillors to take part in semi-structured interviews to explore their views of accountability for tackling childhood obesity, where accountability is a mechanism of holding actors to account. We aimed to identify factors that could limit or improve accountability in the local context. By focussing specifically on councillors as decision-makers, this study explores if these actors see themselves as having a role as agents or as principals in supporting action on the issue and examines the implications for progressing local obesity prevention policies.

## Data and Methods

### Study design

This qualitative study involved semi-structured interviews with councillors in Southampton City Council in England, which is a unitary authority with an estimated population of 256,000. Unitary authorities are responsible for a range of public services such as education, social care, housing, transport and waste collection. At the time of this study, the council was led by a Labour administration (30 Labour and 18 Conservative councillors). The lead author, a public health practitioner and researcher at the council, sent an email to all 48 Southampton City Council councillors inviting them to participate in interviews to explore their views on accountability for tackling childhood obesity. A follow-up email was sent if no reply was received. Councillors expressing an interest were sent a consent form and information about the study.

Semi-structured interviews were conducted face-to-face or by telephone and were audio-recorded. The audio-recordings were transcribed verbatim. Ethical approval was provided by University of Southampton Faculty of Medicine Ethics Committee (ERGO 46896.A1)(*anonymised)*.

### Theoretical framework

Five mechanisms important for achieving accountability have been described in the existing literature [19] and provide a theoretical framework for this study. These mechanisms (described below) can support optimal governance arrangements for networks involved in addressing an issue. (I) C*lear responsibilities and mandates* should be defined among those with the authority to act. (II) *Transparency* is required in all decision-making processes. (III) *Political oversight* is required from elected officials. (IV) C*itizen control* is required to ensure citizen involvement in the policy process. (V) C*hecks, balances and sanctions* should be in place, describing the consequences for actions and outcomes [19]. This framework developed by Mees and Driessen 2019 was judged appropriate for several reasons: first, it was developed to understand local network governance arrangements which has parallels to the stakeholder networks involved in tackling obesity; second, the authors suggested although developed for climate change, it could be applied to different policy issues, and third, climate change and obesity have similarities in that both issues arise from complex causes and require collective action [20]. The theoretical framework was used to inform the semi-structured interview questionnaire and to categorise the data during data analysis.

### Data analysis

We imported transcripts into NVIVO-12 and conducted codebook thematic analysis, as it provides a structured approach to coding and is considered a pragmatic method for applied research [21, 22]. Although the questions and overall theme categories were pre-conceptualised based on existing evidence (in line with ‘coding reliability’ thematic analysis), new sub-themes were developed through inductive analysis based on the interpretation of the data by the lead researcher [21]. We categorised data deductively into the five categories of the framework [19]. Under each category, codes were created inductively as new concepts were identified. The coding frame was developed iteratively to best represent the data. Two transcripts were double-coded by a second researcher to support a reflexive analysis through discussion. The coding frame was revised and all transcripts were then coded by the lead researcher using the revised coding frame. Codes representing similar concepts were combined and organised into key sub-themes. The sub-themes that were developed were discussed regularly with other researchers during the analysis.

## Results

At the time of this study, the city council was controlled by a Labour administration and had 30 Labour councillors and 18 Conservative councillors. Interviews were undertaken with 16 of the 48 councillors (twelve Labour and four Conservative councillors) including councillors involved as panel members of the council’s concurrent Scrutiny Inquiry into tackling childhood obesity. Health overview and scrutiny committees (the Scrutiny Inquiry was a panel of this committee) were introduced to councils in response to the lack of local democratic oversight in healthcare in the National Health Service (NHS), to make the previous Primary Care Trusts and the NHS publicly accountable [23]. Scrutiny Inquiry panels are made up of democratically elected local councillors but cannot include the council’s executive (Cabinet). Interviews (mean length 34 min, range 16–62 min) took place November 2019 to June 2020. The data collection coincided with the start of the COVID-19 pandemic.

### Factors that limit accountability for tackling childhood obesity in local government

Four factors that could limit local government accountability for tackling childhood obesity were identified: (1) a lack of national mandates to influence local action, (2) unachievable targets and ambiguity about responsibilities across the system, (3) constrained political oversight and (4) low citizen engagement.

Participants noted that local government-led action was limited by the absence of a national mandate with no commitments of funding, resources or levers, reducing their ability to intervene locally (#1 in Table 1). In particular, participants noted the need for stronger national powers to influence sectors such as businesses and the food industry (#2 in Table 1). Furthermore, participants noted that local action would be limited without a broader national mandate to address the wider determinants of health, such as income and child poverty.

**Table 1:** Direct quotations from councillors who participated in this study

Citation #	Quotation	References
1	“I think there’s a lot of things we would like to do outside of statutory duties, that we just don’t get to do because we don’t have the money or the staff or the time…”	Participant 16
2	“So it’s one massive marketing campaign after another, and it’s quite sadly become very commercialised and I think somebody higher up, I think it’s got to be national government, needs to say, “You need to cut back on your marketing at certain times”	Participant 3
3	“…if we want to say by […] 2030, more of our children have a healthier weight. It’s a nice ambition, but how do we then turn that into a set of metrics that can be achieved without them becoming very narrowly focused…”	Participant 4
4	“We just want to figure out what we can do, and also what we can’t do, that only national government can do… and not try to do the whole thing”	Participant 15
5	“Especially in the current crisis [Covid-19], it will be the economy and getting people back on their feet… So, we’ve got significant social challenges…, that will become the first thing…”	Participant 15
6	“I think it’s important that we engage at a political level, but I think we have to be realistic about whether politicians can make a difference”	Participant 11
7	“This is me going to be extremely cynical but yes if something is more on the public radar, a politician would probably be asking more questions”.	Participant 14
8	We have to stop silo working and accountability breaking down or at least action breaking down simply because, you know, departments in various public sector organisations don’t sufficiently talk to each other”	Participant 12
9	“It’s not so much a case of transparency but a case of communication… So, I mean, the few of us that are on the Scrutiny Inquiry will know what’s happening, but the other members [councillors] are quite a good way of letting communities know what’s happening”	Participant 1
10	“So it’s down to the Cabinet member who has sanctioned a particular role to get it done, and follow that through”	Participant 3
11	“Some of that is to do with I suppose the democratic infrastructure of the council. Just in assuring that actually, it is rightly recognised, where certain reports and certain activities need to be put in front of particular members of the Cabinet, and sometimes the whole Cabinet”	Participant 10
12	“… if as part of that we build in … a feedback or performance measure, however you want to process it, then we will have an obligation to ensure you’re on track”	Participant 11

A key concern for councillors was setting unachievable targets and agreeing metrics for an issue many knew had a complex causal pathway (#3 in Table 1)*.* This was exacerbated by their awareness that local government has a role, but that action is needed across other sectors and levels of government. This added to a lack of clarity as to where accountability lay for different parts of the system (#4 in Table 1).

Participants reported that political oversight of childhood obesity is often constrained by competing priorities, including other public health issues, the duty to promote economic prosperity, and managing the COVID-19 crisis (#5 in Table 1). Several councillors reported that they did not see a role for themselves in tackling childhood obesity, perceiving it as an individual responsibility. Various concerns prevented political oversight, including uncertainty about what could be done, facing criticism if the problem did not improve, and concerns about whether councillors could make an impact (#6 in Table 1). Interviewees highlighted the need for clearer communication about the urgency of tackling childhood obesity and the financial and wider consequences to the council of not acting.

Citizen involvement was considered important and could be promoted through existing mechanisms including Health Watch (an independent organisation to improve health and social care), People’s Panels and citizen assemblies. Participants reported that citizen engagement could be strengthened by the council and other public sector organisations and by raising awareness of the issue. However, participants felt there was a lack of interest among the community for tackling childhood obesity as it has been normalised or people did not think it was relevant for them (#7 in Table 1). There was a view that issues that concerned the public would automatically be present on local government agendas.

### Factors that improve accountability for tackling childhood obesity in local government

The analysis identified four factors which could improve accountability for tackling childhood obesity in local government: (1) the council can set a local mandate for joint working, (2) the council can facilitate sharing of good practice between organisations, (3) officer engagement to keep the issue on the agenda, (4) regular review demanded by leaders across the system.

Despite the lack of a national mandate for local action, participants acknowledged the importance of the council setting a *local* mandate. In this way, the council could act as an enabler for council departments and other public sector organisations to work together and use the levers at their disposal to implement policies and interventions to reduce childhood obesity (#8 in Table 1).

Participants recognised the importance of transparency and sharing data to identify the good practice and areas for improvement. However, most participants did not feel that there was a lack of transparency in decision-making processes. They noted that the council and councillors had a role to facilitate information sharing with communities and between organisations to help ensure resources were being used appropriately (#9 in Table 1). The analysis indicated that political oversight could be increased by an interested Cabinet member with the time and experience to ask the right questions. By asking questions in public fora and meetings about programmes or services, councillors can help to prioritise the issue and keep it on the agenda (#10 in Table 1). However, participants highlighted the important role officers (those responsible for implementing policies and decisions made by local councillors [24]) have in using available council mechanisms and the democratic infrastructure to get the issue onto meeting agendas. This is through the submission of relevant reports and programme/service updates to help to secure political oversight. This included the Health Scrutiny Inquiry process and oversight from the designated Cabinet member, or through processes that involved the whole Cabinet (#11 in Table 1). Participants provided examples of how leaders and institutions could support informal checks, balances and sanctions in the system. These included leaders of other organisations, the political opposition and other councillors’ demanding information on progress for tackling childhood obesity, (#12 in Table 1)*.* This would ensure that policies and actions are reviewed at a senior level encouraging closer scrutiny and monitoring.

## Discussion

Childhood obesity is a global health challenge requiring national governments to invest in population-wide policies. Regional and local governments have a crucial role in using their powers to influence the local infrastructure, urban environments and deliver targeted community interventions. But the issue may not be a priority due to competing pressures and limited budgets. Accountability requires an actor to justify their actions on an issue. Understanding the views of local elected officials on their accountability for tackling childhood obesity may have implications for public health officers implementing local childhood obesity prevention policies. Sixteen local councillors were interviewed from a local council in England to identify the factors which limit and improve accountability for tackling childhood obesity.

This study found that for councillors the role of the council was undermined by limited national action on wider societal factors associated with obesity, including the marketing of unhealthy food. Whilst they did not see a role for themselves individually, councillors did see a role for the council within its functions and powers to set a local mandate for departments and sectors to work together. A concern noted by many councillors was the fear of giving the impression of ‘telling people how to live their lives’ and the issue being an individual responsibility, which may have contributed to the reluctance of being involved individually. This suggests councillors understood that the issue should be addressed, but councillor support may not be volunteered.

Councillors noted that political oversight was limited due to low citizen engagement as it was not perceived to be a ‘vote winning’ issue. However, councillors acknowledged that oversight from a Cabinet member and progress demanded by other system leaders could strengthen accountability for the issue. Our study supports previous research on the importance of a political champion for accountability and keeping an issue on the policy-making agenda [25–28]. However, given the challenges of securing more visible oversight from councillors, the present study suggests that councillors see a role for public health officers in keeping the issue on the agenda of local governance structures to sustain political oversight.

Political actors are accountable to the electorate and policy change can result from a population demanding progress on an issue [29]. Accountability could be further strengthened by ‘peer accountability’ with the threat of losing credibility and through civil society organisations holding government to account [30, 31]. Our findings highlight that a demand for information on progress towards tackling childhood obesity from the political opposition and leaders across the local system, is likely to lead to oversight from councillors. Local system leaders were considered to have an active role to play by calling councillors to account in public forums and potentially providing an informal “check and balance” in the system. Public health officers could assist through engaging citizen representatives and system leaders, in particular the Integrated Care Boards (statutory organisations responsible for managing the NHS budget and improving population health) may provide such an opportunity. More research is required to understand how system leaders can work within local governance structures to influence childhood obesity prevention policies.

This study highlighted that the complex nature of childhood obesity was understood by councillors, and whilst monitoring was considered important, unachievable targets and difficulties in defining responsibilities for different parts of the system limited engagement with the issue. Studies have found that tools such as dashboards for monitoring action can support transparency between partners to review progress and identify gaps [32, 33]. However, other scholars have argued that transparency alone as a mechanism masks complexities by reducing reporting to simple indicators, whereas intelligent accountability is a reflexive and reciprocal process to promote shared learning [34]. The present study suggests that public health officers should carefully identify and agree a mix of targets and indicators with leaders and political leaders, and that not doing so risks reducing political oversight.

In practice, at local authority level, to improve accountability several agent and principal/forum relationships may be necessary [16]. For example, in addition to the officer being accountable to the elected councillor for progress on tackling childhood obesity, the elected councillor could be required to report to colleagues (and other system leaders), to create a chain of accountability to support resource allocation and policy implementation [31].

### Strengths and Limitations 

The study sample was limited to councillors within Southampton City Council, England. However, a third of all councillors from the two political parties were interviewed, providing a wide representation of views. Interviews took place as a Scrutiny Inquiry into tackling childhood obesity was underway; it is likely that only those with an interest in the issue took part and therefore findings may not be transferable to other councils. However, contextual information has been provided to allow practitioners and researchers to consider how the findings may apply to their context. A limitation of this study is that differences in views according to political affiliations were not considered during the analysis; taking into account differences by political party may have revealed further insights into local government accountability for tackling childhood obesity. Interviews were undertaken by a researcher who also worked as a public health officer at the council with a portfolio that included healthy weight. This background insight underpinned their PhD research questions, which aimed to inform public health practice. Participants were aware of this ‘partial’ insider status which relates to working in the same organisation (rather than sharing multiple identities and roles with participants) and this may have led to more detailed responses [35]. However, the public health officer had not previously worked with participants, as engagement with elected members is undertaken at a senior level, normally led by the Director of Public Health. This said, it is not possible to completely exclude some biases in data collection and interpretation arising from the researcher’s partial insider status. The framework used in the study may have limited the analysis if other relevant accountability mechanisms could be applied. However, both a deductive and inductive approach to the analysis were adopted to generate novel insights from the data. Strengths of the study were that the study followed guidelines on rigorous qualitative research, and reflexive practice was undertaken through discussions with collaborators during the analysis.

The data were collected at the beginning of the COVID-19 pandemic and before the release of the national strategy for obesity (published in response to the increased risks experienced by those with COVID-19 who were obese) [36]. Therefore, data relate to a period where infection control and local financial recovery were prioritised. However, given that obesity remains an ongoing policy issue, the findings from this study are expected to be applicable to current local public health policy and practice.

## Conclusions

By viewing childhood obesity prevention through the lens of accountability mechanisms, this study suggests various factors that could improve local government accountability for the issue. Local government councillors saw the importance of using council functions and powers to address the issue, where they could potentially have a role as ‘principal’ in setting a mandate and holding officers to account. Engaging local system leaders could also strengthen accountability where councillors could have the role of ‘agents’ reporting to other system leaders to support policy development and implementation. Future research should explore the role that wider system leaders have as part of local policy-making processes for tackling childhood obesity.

## Data Availability

Taheem, Ravita (2022) Dataset to support the Southampton Doctoral Thesis ‘Optimising local government policy for tackling childhood obesity’. University of Southampton 10.5258/SOTON/D2338 [Dataset].
